# The computer-assisted interview In My Shoes can benefit shy preschool children's communication

**DOI:** 10.1371/journal.pone.0182978

**Published:** 2017-08-15

**Authors:** Karin Fängström, Raziye Salari, Maria Eriksson, Anna Sarkadi

**Affiliations:** 1 Child Health and Parenting (CHAP), Department of Public Health and Caring Sciences, Uppsala University, Uppsala, Sweden; 2 Department of Social Sciences, Ersta Sköndal Bräcke University College, Stockholm, Sweden; TNO, NETHERLANDS

## Abstract

Interviewing children is a cognitively, socially, and emotionally challenging situation, especially for young and shy children. Thus, finding methods that aid rapport and increase these children’s communication is important. The present study investigated whether children’s verbal and non-verbal communicative behavior developed differently during the rapport phase, depending on whether children were situationally shy or not, and whether the interview was conducted using the computer-assisted interview In My Shoes (IMS) or a Standard verbal interview. The sample consisted of 60 children aged 4 to 5-years-old. The results showed that for the shy children in the IMS group their talkativeness increased and their answer latency decreased including the amount of encouragement the child needed to talk, while no changes were observed for the shy children in the Standard verbal interview group. There were no significant differences in the non-verbal behavior for the shy children regardless of the interview method used. For the non-shy children, overall, the interview method did not affect either the verbal or the non-verbal outcomes. Our findings indicate that IMS can be a useful tool during the rapport-building phase with shy children as it helps these children to improve their verbal communication.

## Introduction

For children, an interview is usually a novel situation with an unknown adult which requires the child to both interact with the adult and to try to understand and navigate the scope of the interview. The performance in an interview situation depends on, for example, the child’s ability to understand the questions posed to them, if she or he comprehends the intention of the question as well as knowing what and how much information one is expected to share [1, 2]. The high social demands and sometimes incomprehensible tasks can invoke anxiety and stress in the child [3]. The stress might have a negative influence on the cognitive abilities needed to share memories and experiences and interfere with the child’s capacity to regulate their emotions and attention [4, 5]. For younger children, the situation can be even more demanding as their language and narrative skills are still developing and their memory retrieval strategies and capacities are limited [6, 7].

Thus, in most types of interviews with children, it is important to create an atmosphere where the child feels relaxed and safe to talk about him or herself, i.e., it is important to build rapport with the child through a supportive and child-centered interaction [8–10]. In research, psychotherapy, counseling, and psychological assessment involving children, rapport is considered crucial to facilitate communication [11–15]. In child forensic and child protective interviews, there is often a discrete phase at the beginning of the interview, before the substantive phase starts, targeted at building rapport, to further emphasize its significance [1, 16, 17].

It is important to note that creating a comfortable environment where the child is calm and willing to talk is not only essential during a particular stage of the interview, but rather a process of interaction [5, 18]; however, what happens at the beginning of the interview lays the foundation for its continuation. For example, a study by Collins, Doherty-Sneddon and Doherty [19] found that child protection practitioners clearly perceived rapport-building as an ongoing communicative process. During this process, the adult assessed the child’s cognitive, emotional, and communicative abilities and then adjusted the interview approach according to the assessment. Their aim was to facilitate the communication by reducing the child’s anxiety level and making the child feel comfortable, for example, more time and effort could be needed to achieve this with a shy and worried child. The importance of identifying uncommunicative or reluctant children early in the interview was also recognized by Katz and colleagues [20]. In particular, by being attentive to the non-verbal signs of reluctance, the interviewers could decide whether more time and effort was needed for rapport-building.

### Shy children

Children who exhibit shy or slow-to-warm-up characteristics react to new people, situations, or contexts with vigilant behaviors and motor quieting; moreover, they are verbally quiet and emotionally reserved [21, 22]. In an interview situation, with an unknown adult, the shy child often behaves in an uncommunicative, anxious, or uncooperative manner [9, 23, 24]. Studies have also shown that shy children are less likely to perceive the adult’s efforts to build rapport positively, and the adults themselves are less likely to exhibit rapport-promoting behaviors. An example is that instead of increasing their efforts to support the child, the interviewers become unsupportive and ask fewer open-ended questions, consequently, increasing the child’s resistance [23, 24]. This is problematic since a well conducted rapport- building may, in fact, help children who seem reluctant or uncommunicative at the beginning of the interview to start to talk and open up later [17].

### Techniques in building rapport

To overcome the challenges in creating a positive interaction and an atmosphere where the child feels safe and relaxed, many researchers and clinicians recommend using different forms of techniques, such as age appropriate play, drawing or playing a game [11, 25, 26]. Other potential techniques for building rapport are using a puppet as in the Berkley Puppet Interview [15], a visual tool such as the Life Story Board [27], or a computer assisted interview approach as in In My Shoes or the Bubble Dialogue [28, 29]. Using such techniques may be even more important for young shy children with limited verbal responses, as the interview then not only relies on the children’s verbal abilities, but also gives them the opportunity to answer in their own preferred fashion, for example, by pointing or showing [15].

The use of computer-assisted approaches when conducting interviews or assessments with children and adolescents has increased over the past few years and these methods potentially entail several important benefits. The computer is an enjoyable tool that can engage and maintain children’s motivation and attention [30]. The shared focus on the computer may lessen the social demands of the interview situation [31–33]. Furthermore, the use of a computer reduces the pressure of direct eye contact, which can help children to relax and feel at ease, thus, facilitating both rapport and communication [28, 30, 34]. Computer assisted approaches, for example, the In My Shoes computer assisted interview, is being used in a wide variety of contexts, ranging from hospitals [35] to schools [34], and also by therapists and social workers [36, 37] as well as by forensic psychologists [38].

Only a few studies have examined the use of computers in conducting interviews with young children. These studies [33, 39–41] have demonstrated that children who were interviewed using a computer-assisted approach communicated their experiences with great detail, depth, and accuracy, similar to children who were interviewed using standard face-to-face methods. However, no study has yet examined whether all young children benefit equally well from being interviewed using a computer or whether certain groups of children, for example, shy children for whom the interview presents a demanding and uncomfortable situation, respond better to a computer-assisted approach than to a standard face-to-face one.

The current study was born out of the clinical experiences from conducting interviews with preschool aged children, with and without a computer-assisted interview approach and seeing how the children reacted to the novel interview situation. Some children talked freely and seemed fairly relaxed, while others showed explicit signs of discomfort and were quiet. The clinicians wondered whether the computer-assisted approach was more beneficial to the second group of children since the interview situation seemed to present a more demanding and uncomfortable situation for them.

### Aim

The aim of the present study was to investigate whether verbal and non-verbal communicative behavior developed differently from the beginning of the rapport phase to the start of the substantive phase, depending on whether children were situationally shy or non-shy and whether the interview was conducted using a computer-assisted interview approach or a verbal interview approach. Our hypothesis was that shy children would increase their communication more when interviewed using the computer-assisted approach compared to the standard verbal interview, while the non-shy children would not differ regardless of which method was used. The two interview approaches compared were the computer-assisted interview In My Shoes (IMS) and a slightly modified version of the National Children’s Advocacy Center Child Forensic Interview Structure, termed the Standard verbal interview, throughout the paper.

## Method

### Participants and procedure

Families with children aged 4 or 5-years-old attending their annual health check up at the Child Health Center (CHC), were invited to participate in the study. There were no exclusion criteria except age. The recruitment took place over a 20-month period between 2013 and 2015. The CHCs were situated in five areas, with varying socio-demographic characteristics in two larger municipalities in Sweden. The annual health visits are a part of the services provided by the CHCs in Sweden, which are utilized by 99 percent of parents of children up to age six [42]. For those families giving their written consent to participate, the nurse video-recorded the visit. Eighty families agreed to participate in the study; however, 11 children were never interviewed: four because the families withdrew their consent and seven because of an administrative mistake. We conducted 69 interviews in total, though two interviews were not recorded. Out of the 67 video-recorded interviews, all children that talked about the visit to the CHC were included. The final sample thus included 60 children (50% female), whereof 31 were 4-year-olds and 29 were 5-year-olds. The children were predominantly from families with highly educated parents (81.7% university level) who were born in Sweden (83.3%). All children went to a regular preschool.

The children were randomized to be interviewed about their visit to the CHC using either the IMS interview or the slightly modified version of the National Children’s Advocacy Center (NCAC) Child Forensic Interview Structure, termed the Standard verbal interview. Out of the 60 interviews included in this study, 30 children were interviewed using IMS (53% 4-year-olds) and 30 children were interviewed using the Standard verbal interview (50% 4-year-olds).

The interviews were conducted at the child’s preschool 2–5 weeks after the CHC visit. Each interview was performed by an interviewer who had a Master of Science degree in sociology or psychology. Both interviewers (one male and one female) were trained and accredited in using the IMS computer-assisted interview as well as the NCAC Child Forensic Interview Structure. All the interviews were video-recorded. Children were asked for verbal assent during the introduction of the interview. They were also informed of their right to end the interview at any stage.

The trial was approved by the Regional Ethical Review Board in Uppsala (Dnr 2012/387). All of the interview data were anonymized to protect the identity of the informants. Families were provided with two written information sheets prior to the visit to the Child Health Center (one for the parents and one for the children). The children were also provided with verbal information about the study at the beginning of the interview as well as information about their right to end the interview at any time. Thus, written consent was obtained from all parents and verbal assent was obtained from all children prior to commencing the recording of interviews. The Swedish Law on Ethics in Human Subjects [43] allows informed consent to be either verbal or in writing.

### Interviewing methods

#### In My Shoes

In My Shoes (IMS) is a computer-assisted interview that can be used as an aid when interviewing children about their experiences and emotions in various settings and with different people. The interviewer uses IMS collaboratively with the child in a triadic conversation where the interviewer, the child, and the computer are part of the three-way process.

IMS consists of a number of interactive modules that give a structure and a scaffold on which to build the interview; however, the modules can be used flexibly to fit the purpose of the interview. The interview is semi-structured, and it opens up areas of conversation. The aim is for the child to self-express. The modules provide different tools, i.e., stylized icons of emotions, people, places, speech, thoughts, and sensations, which can be personalized to the interviewee and be used by the interviewee when communicating his or her experiences. The content of the program thus works both as a prompt for the interviewer when guiding the conversation and as a facilitator for the child to share his or her experiences.

One intended use of the program is when interviewing children about suspected abuse; thus, there is a need for high quality forensic information. Both the questions and the icons have been developed to adhere to these requirements [44].

The first modules render an assessment of the child’s emotional literacy and facilitate rapport-building. By working through the modules together and having the focus on the computer, the social demands of the interview situation are reduced; accordingly, children can relax and build rapport, which in turn can enable communication and sharing of information [28, 38]. Hence, there is no need for an additional rapport phase of the interview. In the following modules, the focus of the interview is narrowed down to a particular place, the significant people present in that place, and the exploration of the emotions and experiences related to the people in that place.

**Fig 1:**
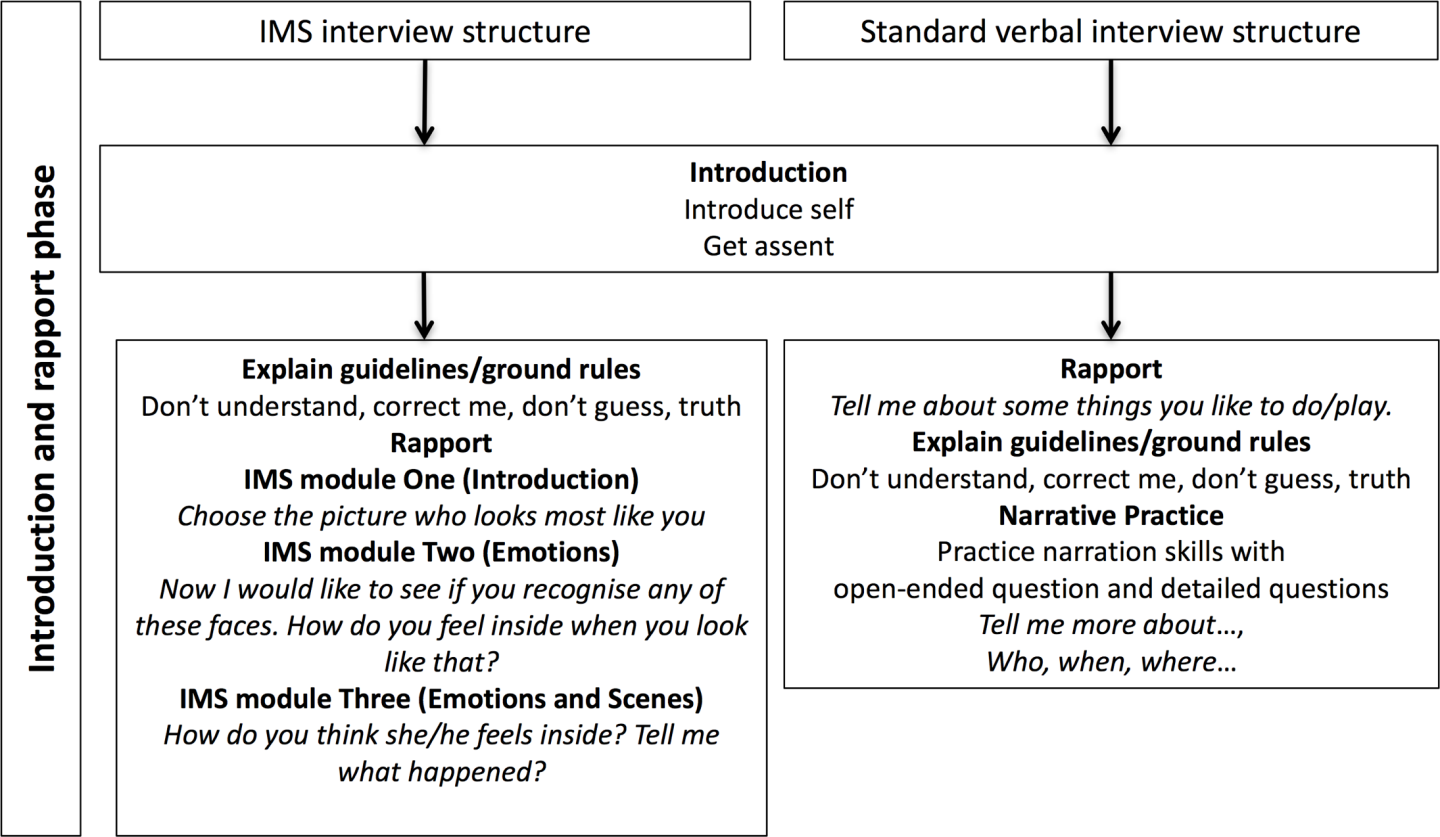
The components of the rapport phase in the two interview structures.

In the current study, the focus was the rapport phase during which the first three modules in IMS were used together with the child (see Fig 1 for an overview of the rapport phase of the two methods). These modules allowed the child to choose a figure as a self-representation; the child named the stylized icons showing different emotions and then practiced using these emotion-faces. Throughout the interview, the interviewer asked mainly open-ended questions. Detailed questions were used when needed to scaffold the interview or acquire more details. Direct questions, for example, yes/no questions or multiple-choice questions, and leading questions were avoided as much as possible. Both interviewers adhered to the interviewing guidelines (Fig 1).

#### Standard verbal interview

A slightly modified version of the NCAC Child Forensic Interview Structure, termed the Standard verbal interview, was used in this study. The NCAC Child Forensic Interview Structure is a method for conducting forensic interviews, created by the National Children’s Advocacy Center (NCAC) in the United States. This interview structure builds upon well-researched components known to help children to share reliable information in a developmentally sensitive way [5, 45, 46]. The method is widely used in the United States as well as in other parts of the world [47].

Similar to the NCAC structure, the Standard verbal interview included three phases: the introduction/rapport, the substantive, and the closure. However, we did not include the phase where the family is discussed, which is included in the NCAC structure. During the rapport phase, the focus was to create a supportive environment and to build rapport (Fig 1). The interviewer asked the child what she or he likes to play and listened in a careful and active way. This was followed by an explanation of the ground rules; thereafter followed a short narrative practice where the child was asked to describe an event more in depth using the recommended types of questions, i.e., open-ended questions and detailed questions when needed. Direct questions and leading questions were avoided.

In both the IMS interviews and the Standard verbal interviews, the substantial phase followed the rapport phase. During the substantial phase, the child’s visit to the CHC was explored; thereafter, in the closure phase, the child was asked about additional information, he or she chose a sticker, and the interviewer thanked the child for participating. The phases of both interview structures have been thoroughly described elsewhere [48].

### Coding

A coding scheme was developed to rate the verbal and non-verbal observable manifestations of social behavior during the interviews. The variables coded included verbal behavior (i.e., talkativeness, answer latency, and the amount of encouragement the child needed to talk) and non-verbal behavior (i.e., facial expression, eye contact, and body tenseness). In previous research, these behaviors have been studied in relation to anxiety, communication, and as signs of shyness in children [49–54].

Each variable was rated on a 5-point scale, where low values indicated shy and uncommunicative behavior and high values indicated non-shy and communicative behavior. Since we were interested in the development of these variables during the rapport phase of the interviews, each interview was coded at the beginning of the rapport phase and at the beginning of the substantive phase (right after the rapport phase was finished). The coding proceeded for two minutes at each time point and the ratings were based on the overall impressions of the child’s behavior during that time (S1 Dataset).

Drawing from the experience of conducting the interviews as well as literature on non-verbal signs of shy behavior, children who, at the beginning of the rapport phase, were very tense in their bodies and gestured very little or not at all, were defined as situationally shy (those who scored a 1 or 2 at the body tenseness variable). Children scoring from 3–5 were defined as situationally non-shy. There were nine children in the IMS condition and eleven children in the Standard verbal condition who were defined as situationally shy.

#### Inter-rater reliability

Three coders, with a master’s degree in psychology or sociology, performed the coding. Two of the coders were the interviewers in the study. The third coder was not involved in conducting the interviews but had previous experience in coding interviews. The coders trained on a set of interviews that were not to be included in the study, until they reached satisfactory agreement. To assess the interrater reliability between the three coders, Krippendorff’s alpha was calculated [55]. Krippendorff’s alpha was chosen because it can be used with several coders, with different scales of measurement, small sample sizes, and with or without missing data [56]. The recommended reliability standard is α ≥ .80 [56, 57], and our analysis showed an α of .84.

### Data analysis

The mean values and standard deviations were calculated for the length in minutes of the rapport phase of the interviews, and a two-way ANOVA and t-tests were performed to analyze any differences between the interview conditions and differences between the levels of shyness.

The main aim of the current study was to investigate whether verbal and non-verbal social behavior developed differently from the beginning of the rapport phase to the start of the substantive phase (interview phase), depending on whether children were situationally shy or non-shy (shyness), and whether the interview was conducted using IMS or Standard verbal interview (interview method). Therefore, to examine the changes in talkativeness, answer latency, the amount of encouragement the child needed to talk, facial expression, and eye contact, we conducted a series of mixed model ANOVAs with one within subject variable (interview phase) and two between subject variables (shyness and interview method). The simple effects were only investigated when the *p* value of the three-way interaction was below .10. The statistical analyses were performed using IBM SPSS Statistics 22.0.

## Results

### Preliminary analysis

A two-way ANOVA was conducted to investigate any interaction between the level of shyness and the interview method on the time spent in the rapport phase. This showed no significant interaction, *F*(3, 56) = 0.950, *p = ns*. Independent sample *t*-tests revealed that there was a significant difference in the time spent in the rapport phase between the interview conditions, *t* (58) = 8.16, *p <* .001, *d* = 2.15. The rapport phase of the IMS interviews (M = 11.32, SD = 2.38) lasted almost twice as long as the Standard verbal interviews (M = 6.06, SD = 2.30). There was no significant difference between the situationally shy children and the non-shy children regarding the time spent in the rapport phase *t* (58) = 0.11, *p = ns* (M = 8.54, SD = 3.45 and M = 8.47, SD = 3.47 for shy and non-shy children, respectively).

### The effect of shyness and interview method on social behavior

The mixed model ANOVAs showed that the three-way interaction (interview phase*shyness*interview method) was significant for talkativeness: *F*(1, 55) = 6.28, *p* = .015, η^2^_*p*_ = 0.10 and answer latency *F*(1, 56) = 4.08, *p* = .048, η^2^_*p*_ = 0.068. In addition, a trend was observed for the amount of encouragement needed *F*(1, 55) = 3.41, *p* = .070, η^2^_*p*_ = 0.058. The three-way interaction was not significant for facial expression *F*(1, 55) = 0.10, *p* = .749, η^2^_*p*_ = 0.002 or eye contact *F*(1, 54) = 0.87, *p* = .355, η^2^_*p*_ = 0.016.

Therefore, the simple effects were investigated for talkativeness, answer latency, and the amount of encouragement needed (Table 1). The results for *talkativeness* showed that while shy children in the IMS group talked more over time (95% CI for mean differences [.85 to 2.04]), no changes were observed for the shy children in the Standard verbal interview group (95% CI for mean differences [-.35 to .72]). Non-shy children in the IMS and Standard verbal interview group showed a similar pattern of change over time, with both groups’ talkativeness increasing during the rapport phase (95% CI for mean differences [.19 to .96] and [.12 to .93] for children in the IMS and Standard verbal interview group, respectively). The results for *answer latency* showed that for the shy children interviewed using the IMS, their answer latency decreased during the rapport phase (95% CI for mean differences [.29 to 1.49]), while the answer latency for the shy children interviewed using the Standard verbal interview did not change over time (95% CI for mean differences [−.63 to .45]). No changes over time were reported for the non-shy children in either of the interview groups (95% CI for mean differences [-.25 to .53] and [-.25 to .57] for children in the IMS and Standard verbal interview group, respectively). The results for the *amount of encouragement needed* showed that the shy children interviewed with the IMS needed less encouragement over time (95% CI for mean differences [.61 to 1.84]), while there was no change over time for the shy children interviewed with the Standard verbal interview (95% CI for mean differences [−.46 to .65]). Non-shy children showed no change over time in either of the interview groups (95% CI for mean differences [-.11 to .71] and [-.32 to .53] for children in the IMS and Standard verbal interview group, respectively).

**Table 1:** Mean scores on verbal social behavior by shyness and interview condition across the two time points.

		*Start of rapport phase*	*Start of substantive phase*	*M difference*	*[95% CI]*	η^*2*^_*p*_
**Talkativeness**						
Shy	IMS	1.89	3.33	1.44*	[0.85, 2.04]	0.75
	Standard verbal	2.73	2.91	0.18	[-0.35, 0.72]	0.04
Non-shy	IMS	3.14	3.71	0.57*	[0.19, 0.96]	0.26
	Standard verbal	3.79	4.32	0.53*	[0.12, 0.93]	0.38
**Answer latency**						
Shy	IMS	3.00	3.89	0.89*	[0.29, 1.49]	0.51
	Standard verbal	3.18	3.09	-0.09	[-0.63, 0.45]	0.00
Non-shy	IMS	4.00	4.14	0.14	[-0.25, 0.53]	0.04
	Standard verbal	4.11	4.26	0.16	[-0.25, 0.57]	0.07
**Encouragement needed**						
Shy	IMS	3.33	4.56	1.22*	[0.61, 1.84]	0.64
	Standard verbal	3.46	3.55	0.09	[-0.46, 0.65]	0.01
Non-shy	IMS	4.30	4.60	0.30	[-0.11, 0.71]	0.13
	Standard verbal	4.53	4.63	0.11	[-0.32, .53]	0.02

* *p* < .05

### Sensitivity analysis

Normality assumption was violated for several of the dependent variables. Transforming these variables did not change their distribution. Therefore, we used the difference between the substantive phase and the rapport phase as the dependent variables in a series of bootstrapped (n = 10,000) two-way ANOVAs. Inspection of the bootstrapped 95% confidence intervals for means revealed that the results remained the same for the variables facial expression, eye contact, talkativeness, and amount of encouragement needed. However, the shyness and interview method interaction was no longer significant for answer latency.

## Discussion

The main aim of the current study was to investigate whether verbal and non-verbal communicative behavior developed differently during the rapport phase, depending on whether children were situationally shy or non-shy, and whether the interview was conducted using the IMS or the Standard verbal interview. The results showed that the talkativeness increased and the answer latency decreased for the shy children interviewed with IMS, while no change was observed for the shy children interviewed with the Standard verbal interview. This pattern was also evident for the amount of encouragement the child needed to talk, with shy children interviewed using the IMS needing less encouragement. There were no significant differences in the non-verbal behavior for the shy children, regardless of the interview method used. For the *non-shy* children, overall, the interview method did not affect either the verbal or the non-verbal outcomes. This indicates that IMS helped situationally shy children to increase their verbal communication, thus, fulfilling the goal of facilitating communication during the rapport-building phase, something that both clinicians and researchers strive for [9, 11, 19]. Another important aspect of this result is the potential benefit for the interviewer, as children who do not communicate tend to increase the risk that the interviewer will give way to unsupportive behavior and poor question techniques [23, 24]. Providing both the interviewer and the interviewee with a tool that can increase children’s communication early on in the interview could be vital for the interaction and an important key for a successful interview.

This study did not set out to investigate why or how the IMS helped children increase their communication; however, one possible explanation could be that using an aid such as the IMS shifts the focus from the child to the computer, which might lessen the social demands of the interview. This could be more beneficial for shy children, as research has demonstrated that self-attention and self-consciousness play a key role in, for example, shyness, embarrassment, and negative affect, in general [58]. The increased self-focus and stress becomes particularly problematic as it may have a negative impact on the child’s cognitive and emotional abilities. With IMS, the child and the interviewer have a joint external task, and the visible structure of the modules could increase the child’s sense of control and mastery, as has been proposed by previous research [28].

We did not find any significant changes in the non-verbal behavior (eye-contact and facial expression) in the interaction between shyness and the interview method. The eye-contact measure was problematic as children in the IMS interviews divided their gaze between the computer and the interviewer. Thus, looking at the computer could be understood both as a sign of engagement in the task and/or as a sign of avoiding to look at the interviewer, something which has been discussed in previous research [24]. This makes the results of eye contact difficult to interpret. The lack of effect on the facial expression variable could be due to the non-verbal behavior not being affected by the interview method to the same extent as the verbal behavior. Another potential explanation could be that the change in behavior is sequential and that it was easier for children to start to talk more, while the non-verbal signs of anxiety, especially in shy children, might change at a slower pace. Investigation of the raw data suggests that this may be a plausible explanation, as we observed slightly more improvement, although not significant, for the shy children in the IMS group compared to their counterparts in the Standard verbal interview group. The size of the sample might have been too small to detect this subtle difference.

The rapport phase of the IMS interviews lasted almost twice as long as the Standard verbal interviews. In the IMS interviews, the child named the icons showing different emotions and then practiced using these emotion-faces, something that took time. It is also built into the IMS method that the child should have control over the pace of the interview. These factors may explain the difference in time between the interview methods. Interviews that take more time are in some contexts perceived as negative, in particular, a lengthy period of rapport building, which might exhaust children’s attentional resources and reduce their productivity in the substantial phase, could be problematic [59, 60]. However, the effect of time on rapport is being discussed and young and shy children might, in fact, need more time to gain trust in the adult and feel comfortable [11]. Therefore, several researchers and clinicians use extended rapport building, assessments, or multiple interviews with these children [18, 19, 61, 62]. When using the IMS, the fact that the interviewer and the child spent more time together could in itself positively affect the child’s behavior. Thus, a tool that allows for more time for the interview while maintaining the child’s interest and motivation could have a beneficial effect on communication.

### Limitations

One limitation of this study is the small sample size, which is a consequence of this study not being planned for specifically and the power being calculated to detect the differences in the other outcomes between the interview methods, such as accuracy and completeness (the reference has been removed to conceal the authors’ identity). Also, because the study was based on the clinical experiences of conducting the interviews with children, no standardized questionnaires were used to measure the children’s temperamental characteristics; moreover, we have no information about how shy the children were in other contexts. The children in our study were at least situationally shy, and the interviewer had to deal with this instantly. The study is thus exploratory, and the results should be interpreted with caution. However, in real life settings, most professionals have to make decisions about how to interview a child based on the child’s overt behavior with little or no information about the child beforehand.

It should also be noted that parents and children self-selected into the study; therefore, families with very shy children might have declined to participate. Future studies are needed to investigate whether an approach like IMS would be even more beneficial for these children.

We have little knowledge about the children’s developmental level, such as their general language ability. The majority of the participating families were highly educated and born in Sweden. However, in all probability, most children were typically developing children because (1) all children were recruited from a general population with no exclusion criteria except age, (2) we actively encouraged the nurses to invite all families that attended the CHC for their child’s annual health check-up, a service used by 99 percent of parents of children up to age six, and (3) all children were attending regular preschools (almost 95 percent of children aged 4–5-years-old attend preschool in Sweden [63]. Nevertheless, more information about children’s specific developmental level would most certainly have added to a more in-depth understanding of their behavior in this specific situation.

There is also a need to investigate other groups of children for whom the interview situation can be especially demanding, for example, children with difficulties in communicating verbally, children with hyperactive behavior, or traumatized children. Furthermore, studies are also needed to explore the content of what is being communicated with the different kinds of methods and if, for example, emotionally laden experiences are more easily communicated with one method or the other.

## Conclusion

The interview situation is a challenging experience for shy children, and conducting interviews with uncommunicative children can be difficult. Our findings indicate that IMS can be a useful tool during the rapport phase with situationally shy children as it increased their talkativeness and decreased their answer latency as well as the amount of encouragement the child needed to talk.

## Supporting information

S1 Dataset Child verbal and non-verbal behavior coded at the two time points. (XLSX)

## References

[1] Hershkowitz I, Lamb ME, Orbach Y, Katz C, Horowitz D. The development of communicative and narrative skills among preschoolers: Lessons from forensic interviews about child abuse. Child Dev. 2012;83(2):611–22. doi: http://dx.doi.org/10.1111/j.1467-8624.2011.01704.x . 22181976 10.1111/j.1467-8624.2011.01704.x

[2] Saywitz KJ, Camparo LB. Interviewing Children: A Primer In: Melton GB, Ben-Arieh A, Cashmore J, Goodman GS, Worley NK, editors. The SAGE handbook of child research. Los Angeles: SAGE Publications Ltd; 2014.

[3] Roebers CM, Schneider W. Individual differences in children's eyewitness recall: The influence of intelligence and shyness. Applied Developmental Science. 2001;5(1):9–20. doi: http://dx.doi.org/10.1207/S1532480XADS0501_2

[4] Carter CA, Bottoms BL, Levine M. Linguistic and socioemotional influences on the accuracy of children's reports. Law Hum Behav. 1996;20(3):335–58. doi: http://dx.doi.org/10.1007/BF01499027

[5] National Board of Health and Welfare. Listening to children in foster care—Eliciting reliable reports from children: Review of influential factors. http://www.socialstyrelsen.se/ 2015 2015-1-17.

[6] Nelson K, Fivush R. The emergence of autobiographical memory: A social cultural developmental theory. Psychol Rev. 2004;111(2):486–511. doi: http://dx.doi.org/10.1037/0033-295X.111.2.486 . 15065919 10.1037/0033-295X.111.2.486

[7] Schwenck C, Bjorklund DF, Schneider W. Developmental and individual differences in young children’s use and maintenance of a selective memory strategy. Dev Psychol. 2009;45(4):1034 doi: http://dx.doi.org/10.1037/a0015597 19586178 10.1037/a0015597

[8] Powell MB, Lancaster S. Guidelines for interviewing children during child custody evaluations. Aust Psychol. 2003;38(1):46–54. doi: http://dx.doi.org/10.1080/00050060310001707017

[9] Sattler JM. Clinical and forensic interviewing of children and families: Guidelines for the mental health, education, pediatric, and child maltreatment fields San Diego: Jerome M Sattler Publisher; 1998.

[10] Almerigogna J, Ost J, Akehurst L, Fluck M. How interviewers’ nonverbal behaviors can affect children’s perceptions and suggestibility. J Exp Child Psychol. 2008;100(1):17–39. doi: http://dx.doi.org/10.1016/j.jecp.2008.01.006 18316091 10.1016/j.jecp.2008.01.006

[11] Irwin LG, Johnson J. Interviewing young children: Explicating our practices and dilemmas. Qual Health Res. 2005;15(6):821–31. doi: http://dx.doi.org/10.1177/1049732304273862 15961878 10.1177/1049732304273862

[12] Karver MS, Handelsman JB, Fields S, Bickman L. Meta-analysis of therapeutic relationship variables in youth and family therapy: The evidence for different relationship variables in the child and adolescent treatment outcome literature. Clin Psychol Rev. 2006;26(1):50–65. doi: http://dx.doi.org/10.1016/j.cpr.2005.09.001 16271815 10.1016/j.cpr.2005.09.001

[13] Leach MJ. Rapport: A key to treatment success. Complement Ther Clin Pract. 2005;11(4):262–5. doi: http://dx.doi.org/10.1016/j.ctcp.2005.05.005 16290897 10.1016/j.ctcp.2005.05.005

[14] Donate-Bartfield E, Passman RH. Establishing rapport with preschool-age children: Implications for practitioners. Child Health Care. 2000;29(3):179–88. doi: http://dx.doi.org/10.1207/S15326888CHC2903_3

[15] Measelle JR, Ablow JC, Cowan PA, Cowan CP. Assessing young children's views of their academic, social, and emotional lives: An evaluation of the self-perception scales of the Berkeley puppet interview. Child Dev. 1998;69(6):1556–76. doi: http://dx.doi.org/10.1111/j.1467-8624.1998.tb06177.x 9914640

[16] Lamb ME, Orbach Y, Hershkowitz I, Esplin PW, Horowitz D. A structured forensic interview protocol improves the quality and informativeness of investigative interviews with children: A review of research using the NICHD Investigative Interview Protocol. Child Abuse Negl. 2007;31(11–12):1201–31. doi: http://dx.doi.org/10.1016/j.chiabu.2007.03.021 ; PubMed Central PMCID: PMCPMC2180422. 18023872 10.1016/j.chiabu.2007.03.021 PMC2180422

[17] Wood JM, McClure KA, Birch RA. Suggestions for improving interviews in child protection agencies. Child Maltreatment. 1996;1(3):223–30. doi: http://dx.doi.org/10.1177/1077559596001003005

[18] National Children's Advocacy Center. National Children’s Advocacy Center’s Child Forensic Interview Structure. Huntsville, Alabama: 2012.

[19] Collins K, Doherty-Sneddon G, Doherty MJ. Practitioner perspectives on rapport building during child investigative interviews. Psychology, Crime and Law. 2014;20(9):884–901. doi: http://dx.doi.org/10.1080/1068316X.2014.888428

[20] Katz C, Hershkowitz I, Malloy LC, Lamb ME, Atabaki A, Spindler S. Non-verbal behavior of children who disclose or do not disclose child abuse in investigative interviews. Child Abuse Negl. 2012;36(1):12–20. doi: http://dx.doi.org/10.1016/j.chiabu.2011.08.006 22265935 10.1016/j.chiabu.2011.08.006

[21] Fox NA, Henderson HA, Rubin KH, Calkins SD, Schmidt LA. Continuity and discontinuity of behavioral inhibition and exuberance: Psychophysiological and behavioral influences across the first four years of life. Child Dev. 2001;72(1):1–21. doi: http://dx.doi.org/10.1111/1467-8624.00262 11280472 10.1111/1467-8624.00262

[22] Kagan J. Temperamental contributions to social behavior. Am Psychol. 1989;44(4):668 doi: http://dx.doi.org/10.1037/0003-066X.44.4.668

[23] Hershkowitz I, Orbach Y, Lamb ME, Sternberg KJ, Horowitz D. Dynamics of forensic interviews with suspected abuse victims who do not disclose abuse. Child Abuse Negl. 2006;30(7):753–69. doi: http://dx.doi.org/10.1016/j.chiabu.2005.10.016 16846642 10.1016/j.chiabu.2005.10.016

[24] Rotenberg K, Eisenberg N, Cumming C, Smith A, Singh M, Terlicher E. The contribution of adults' nonverbal cues and children's shyness to the development of rapport between adults and preschool children. International Journal of Behavioral Development. 2003;27(1):21–30. doi: http://dx.doi.org/10.1080/01650250143000571

[25] Reynolds CR, Kamphaus RW. Handbook of psychological and educational assessment of children: Personality, behavior, and context: Guilford Press; 2003.

[26] Lowenstein L. Favorite therapeutic activities for children, adolescents, and families: Practitioners share their most effective interventions. http://lianalowenstein.com/e-booklet.pdf: Champion Press; 2011 [cited 2016 May 18].

[27] Chase RM, Medina MF, Mignone J. The Life Story Board: A feasibility study of a visual interview tool for school counsellors. Canadian Journal of Counselling and Psychotherapy. 2012;46(3):183–200.

[28] Calam R, Cox A, Glasgow D, Jimmieson P, Groth Larsen S. Assessment and therapy with children: Can computers help? Clin Child Psychol Psychiatry. 2000;5(3):329–43. doi: http://dx.doi.org/10.1177/1359104500005003004

[29] Jones A, Selby C. The use of computers for self-expression and communication. Journal of Computing and Childhood Education. 1997;8(2–3):199–214. .

[30] Steward MS, Steward DS, Farquhar L, Myers JEB, Reinhart M, Welker J, et al. Interviewing young children about body touch and handling. Development MotSfRiC, editor: Monographs of the Society for Research in Child Development; 1996. i-232 p. 8972588

[31] Powell MB, Wilson CJ, Hasty MK. Evaluation of the usefulness of ‘Marvin’; a computerized assessment tool for investigative interviewers of children. Comput Human Behav. 2002;18(5):577–92. doi: http://dx.doi.org/10.1016/S0747-5632(02)00003-1

[32] Roebers CM, Howie P, Beuscher E. Can private reports enhance children’s event recall, lower their suggestibility and foster their metacognitive monitoring compared to face-to-face interviews? Comput Human Behav. 2007;23(1):749–69. doi: http://dx.doi.org/10.1016/j.chb.2004.11.007

[33] Bokström P, Fängström K, Calam R, Lucas S, Sarkadi A. ‘I felt a little bubbly in my tummy’: Eliciting pre‐schoolers' accounts of their health visit using a computer‐assisted interview method. Child Care Health Dev. 2016;42(1):87–97. doi: http://dx.doi.org/10.1111/cch.12293 26564782 10.1111/cch.12293

[34] Barrow W, Hannah EF. Using computer-assisted interviewing to consult with children with autism spectrum disorders: An exploratory study. Sch Psychol Int. 2012;33(4):450–64. doi: http://dx.doi.org/10.1177/0143034311429167

[35] Calam RM, Jimmieson P, Cox AD, Glasgow DV, Groth Larsen S. Can computer-based assessment help us understand children’s pain? Eur J Anaesthesiol. 2000;17:284–8. doi: http://dx.doi.org/10.1097/00003643-200005000-00002 10926067 10.1046/j.1365-2346.2000.00655.x

[36] Bøhren IE, Stabrun R, Tjersland OA. "De har litt sånn bob-bob-stemme". En studie av «In My Shoes» brukt i barnesamtaler ved skilsmissekonflikter. Fokus på Familien,. 2014;(4):273–94.

[37] Cousins J, Simmonds J. Investigating the involvement of disabled children in using In My Shoes as a family-finding tool: A pilot project. Adoption and Fostering. 2011;35(4):4–19. doi: http://dx.doi.org/10.1177/030857591103500402

[38] Grasso F, Atkinson K, Jimmieson P, editors. In My Shoes—a computer assisted interview for communicating with children about emotions. 2013 Humaine Association Conference on Affective Computing and Intelligent Interaction (ACII); 2013; Geneva, Switzerland: IEEE.

[39] Powell MB, Wilson JC, Thomson DM. Eliciting children’s recall of events: how do computers compare with humans? Comput Human Behav. 2002;18(3):297–313. doi: http://dx.doi.org/10.1016/S0747-5632(01)00045-0

[40] Donohue A, Powell MB, Wilson JC. The effects of a computerised interview on children's recall of an event. Comput Human Behav. 1999;15(6):747–61. doi: http://dx.doi.org/10.1016/S0747-5632(99)00045-X

[41] Fängström K, Bokström P, Dahlberg A, Calam R, Lucas S, Sarkadi A. In My Shoes–Validation of a computer assisted approach for interviewing children. Child Abuse Negl. 2016;58:160–72. doi: http://dx.doi.org/10.1016/j.chiabu.2016.06.022 27394051 10.1016/j.chiabu.2016.06.022

[42] Wallby T. Lika för alla?: Social position och etnicitet som determinanter för amning, föräldrars rökvanor och kontakter med BVC. Uppsala: Uppsala University; 2012.

[43] Lag om etikprövning av forskning som avser människor [Swedish law on ethical review of research involving humans] (SFS 2003:460) (2003).

[44] Joyce B, Glasgow D. In My Shoes: interactive computer assisted interview In: The British Association for the Study and Prevention of Child Abuse and Neglect (BASPCAN), editor. Keeping children safe in an uncertain world: Learning from evidence and practice; Belfast: BASPCAN; 2012.

[45] National Children's Advocacy Center. Update to National Children’s Advocacy Center’s Child Forensic Interview Structure. Huntsville, Alabama: 2014.

[46] Faller K. Forty years of forensic interviewing of children suspected of sexual abuse, 1974–2014: Historical benchmarks. Social Sciences. 2015;4(1):34–65. doi: http://dx.doi.org/10.3390/socsci4010034

[47] National Children's Advocacy Center. National Children's Advocacy Center: History http://www.nationalcac.org/table/about/history/2015 [cited 2016 September 12]. Available from: http://www.nationalcac.org/table/about/history/.

[48] Fängström K, Bokström P, Dahlberg A, Calam R, Lucas S, Sarkadi A. In My Shoes–Validation of a computer assisted approach for interviewing children. Child Abuse Negl. 2016;58:160–72. doi: http://dx.doi.org/10.1016/j.chiabu.2016.06.022 27394051 10.1016/j.chiabu.2016.06.022

[49] Glennon B, Weisz JR. An observational approach to the assessment of anxiety in young children. J Consult Clin Psychol. 1978;46(6):1246–57. doi: http://dx.doi.org/10.1037/0022-006X.46.6.1246 730875 10.1037//0022-006x.46.6.1246

[50] van Brakel AM, Muris P, Bögels SM. Relations between parent-and teacher-reported behavioral inhibition and behavioral observations of this temperamental trait. J Clin Child Adolesc Psychol. 2004;33(3):579–89. doi: http://dx.doi.org/10.1207/s15374424jccp3303_15 15271615 10.1207/s15374424jccp3303_15

[51] Dougherty LR, Bufferd SJ, Carlson GA, Dyson M, Olino TM, Durbin CE, et al Preschoolers' observed temperament and psychiatric disorders assessed with a parent diagnostic interview. J Clin Child Adolesc Psychol. 2011;40(2):295–306. doi: http://dx.doi.org/10.1080/15374416.2011.546046 21391025 10.1080/15374416.2011.546046 PMC3063122

[52] Vreeke LJ, Muris P, Mayer B, Huijding J, Bos AE, van der Veen M, et al The assessment of an inhibited, anxiety-prone temperament in a Dutch multi-ethnic population of preschool children. Eur Child Adolesc Psychiatry. 2012;21(11):623–33. doi: http://dx.doi.org/10.1007/s00787-012-0299-0 22790233 10.1007/s00787-012-0299-0 PMC3493658

[53] Bohlin G, Hagekull B, Andersson K. Behavioral inhibition as a precursor of peer social competence in early school age: The interplay with attachment and nonparental care. Merrill-Palmer Quarterly. 2005;51(1):1–19. doi: http://dx.doi.org/10.1353/mpq.2005.0001

[54] Eriksson M, Näsman E. Interviews with children exposed to violence. Children and Society. 2012;26(1):63–73. doi: http://dx.doi.org/10.1111/j.1099-0860.2010.00322.x

[55] Krippendorff K. Reliability in content analysis. Human Communication Research. 2004;30(3):411–33. doi: http://dx.doi.org/10.1111/j.1468-2958.2004.tb00738.x

[56] Hayes AF, Krippendorff K. Answering the call for a standard reliability measure for coding data. Communication methods and measures. 2007;1(1):77–89. doi: http://dx.doi.org/10.1080/19312450709336664

[57] Nunnally JC. Psychometric theory 2nd ed. New York: McGraw-Hill; 1978.

[58] Crozier WR, Alden LE. The essential handbook of social anxiety for clinicians London: John Wiley & Sons; 2005.

[59] Hershkowitz I. Socioemotional factors in child sexual abuse investigations. Child Maltreatment. 2009;14(2):172–81. doi: http://dx.doi.org/10.1177/1077559508326224 19047478 10.1177/1077559508326224

[60] Teoh Y-S, Lamb ME. Interviewer demeanor in forensic interviews of children. Psychology, Crime & Law. 2013;19:145–59.

[61] Faller KC, Cordisco-Steele L, Nelson-Gardell D. Allegations of sexual abuse of a child: What to do when a single forensic interview isn't enough. Journal of Child Sexual Abuse. 2010;19(5):572–89. doi: http://dx.doi.org/10.1080/10538712.2010.511985 20924911 10.1080/10538712.2010.511985

[62] Carnes CN, Wilson C, Nelson-Gardell D. Extended forensic evaluation when sexual abuse is suspected: A model and preliminary data. Child Maltreatment. 1999;4(3):242–54. doi: http://dx.doi.org/10.1177/1077559599004003005 10.1177/1077559501006003004 11471630

[63] The Swedish National Agency for Education. Barn och grupper i förskolan 2016 [Children and groups in the preschool 2016] https://www.skolverket.se2017/ [cited 2017 2nd June]. Available from: https://www.skolverket.se/statistik-och-utvardering/statistik-i-tabeller/forskola/barn-och-grupper/barn-och-grupper-i-forskolan-15-oktober-2016-1.260075.

